# Mapping of Transcription Termination within the S Segment of SFTS Phlebovirus Facilitated Generation of NSs Deletant Viruses

**DOI:** 10.1128/JVI.00743-17

**Published:** 2017-07-27

**Authors:** Benjamin Brennan, Veronica V. Rezelj, Richard M. Elliott

**Affiliations:** MRC—University of Glasgow Centre for Virus Research, Glasgow, Scotland, United Kingdom; University of Southern California

**Keywords:** nonstructural protein, phlebovirus, reporter virus, SFTSV, emerging pathogen, transcriptional regulation

## Abstract

SFTS phlebovirus (SFTSV) is an emerging tick-borne bunyavirus that was first reported in China in 2009. Here we report the generation of a recombinant SFTSV (rHB29NSsKO) that cannot express the viral nonstructural protein (NSs) upon infection of cells in culture. We show that rHB29NSsKO replication kinetics are greater in interferon (IFN)-incompetent cells and that the virus is unable to suppress IFN induced in response to viral replication. The data confirm for the first time in the context of virus infection that NSs acts as a virally encoded IFN antagonist and that NSs is dispensable for virus replication. Using 3′ rapid amplification of cDNA ends (RACE), we mapped the 3′ end of the N and NSs mRNAs, showing that the mRNAs terminate within the coding region of the opposite open reading frame. We show that the 3′ end of the N mRNA terminates upstream of a 5′-GCCAGCC-3′ motif present in the viral genomic RNA. With this knowledge, and using virus-like particles, we could demonstrate that the last 36 nucleotides of the NSs open reading frame (ORF) were needed to ensure the efficient termination of the N mRNA and were required for recombinant virus rescue. We demonstrate that it is possible to recover viruses lacking NSs (expressing just a 12-amino-acid NSs peptide or encoding enhanced green fluorescent protein [eGFP]) or an NSs-eGFP fusion protein in the NSs locus. This opens the possibility for further studies of NSs and potentially the design of attenuated viruses for vaccination studies.

**IMPORTANCE** SFTS phlebovirus (SFTSV) and related tick-borne viruses have emerged globally since 2009. SFTSV has been shown to cause severe disease in humans. For bunyaviruses, it has been well documented that the nonstructural protein (NSs) enables the virus to counteract the human innate antiviral defenses and that NSs is one of the major determinants of virulence in infection. Therefore, the use of reverse genetics systems to engineer viruses lacking NSs is an attractive strategy to rationally attenuate bunyaviruses. Here we report the generation of several recombinant SFTS viruses that cannot express the NSs protein or have the NSs open reading frame replaced with a reporter gene. These viruses cannot antagonize the mammalian interferon (IFN) response mounted to virus infection. The generation of NSs-lacking viruses was achieved by mapping the transcriptional termination of two S-segment-derived subgenomic mRNAs, which revealed that transcription termination occurs upstream of a 5′-GCCAGCC-3′ motif present in the virus genomic S RNA.

## INTRODUCTION

The recently reclassified Bunyavirales is an order containing over 350 named virus isolates that are classified into 9 families of viruses, containing 13 genera. In the 2016 release of the International Committee on Taxonomy of Viruses, the Phlebovirus genus is now classified within the Phenuiviridae family (https://talk.ictvonline.org/taxonomy). All phleboviruses share a genome structure that comprises three segments of negative-sense or ambisense RNA (1, 2). The viral genome is composed of the small (S), medium (M), and large (L) RNA segments. The S segment encodes the nucleocapsid (N) protein, the M segment encodes the precursor for the viral glycoproteins (Gn and Gc), and the L segment encodes the viral RNA-dependent RNA polymerase (RdRp). Some viruses within the genus also encode nonstructural proteins within their S or M segments (3).

The phleboviral S segment utilizes an ambisense coding strategy to express the N and nonstructural (NSs) proteins separated by an untranslated intergenic region (IGR). The N protein is translated from a subgenomic mRNA transcribed from the genomic RNA, while the NSs protein is translated from a subgenomic mRNA transcribed from the antigenomic RNA (4, 5). Viral mRNA transcripts contain a 5′ cap structure that is obtained from host cell mRNAs through a cap-snatching mechanism, facilitated through the utilization of an endonuclease domain present within the N-terminal region of the RdRp (6, 7). Previous work confirmed that unlike cellular mRNAs, phlebovirus-derived mRNAs are not polyadenylated at their 3′ ends (8) and are much smaller in size than the genomic or antigenomic RNA from which they are transcribed (9–11). Work by numerous groups has mapped the 3′ end of the N and NSs mRNAs for several viruses within the Phlebovirus genus, such as Rift Valley fever phlebovirus (RVFV) (11–13), sandfly fever Sicilian virus (SFSV) (12), Toscana virus (TOSV) (12, 14), Punta Toro phlebovirus (PTV) (8), and Uukuniemi phlebovirus (UUKV) (6). These data demonstrated that the mRNAs derived from the S segment overlapped with each other and that the 3′ ends of the mRNAs mapped to regions within or just flanking the IGR. It was found for RVFV that transcription termination of both the N and NSs mRNA occurred 3 to 4 nucleotides (nt) upstream of a 5′-(G/A)CUGC_1–3_-3′ motif present in the IGR of the RVFV S segment (11, 12). Lara et al. refined this termination signal sequence motif to be 5′-GCUGC-3′. The latter work also further demonstrated that some sequence variation is tolerated to maintain the termination function of the motif, with 5′-GCAGC-3′ identified as being the sequence responsible for the transcriptional termination of the RVFV L segment (13).

Severe fever with thrombocytopenia syndrome virus (SFTSV), or SFTS phlebovirus, is a novel tick-borne phlebovirus that emerged in China in 2009 (15). The virus caused severe disease in humans, with patients presenting with thrombocytopenia, hemorrhagic manifestations, and multiorgan failure (15, 16), with a case fatality rate ranging from 12 to 15%. Both infectious virus and viral RNA have been isolated from Haemaphysalis longicornis ticks, while viral RNA only has been isolated from Riphicephalus microplus ticks collected in China (15, 17). Retrospective testing and active surveillance of patients presenting with SFTS-like symptoms led to the identification of the virus in Japan (18) and South Korea (19–21). Other tick-borne phleboviruses have also been recently discovered and assigned within the Phlebovirus genus, with a broad global distribution and a range of pathogenicity associated with infection of humans (3). Work rapidly focused on establishing the mechanisms by which SFTSV could cause such severe disease. SFTSV NSs was characterized as a potent antagonist of the mammalian interferon (IFN) response (targeting both induction and signaling pathways) (22–27). Various studies have indicated that as seen with other bunyaviruses, SFTSV NSs is a potent IFN antagonist and could potentially be a major determinant of virulence (28–32).

Reverse genetics systems for the recovery of recombinant phleboviruses such as RVFV have played a crucial role in understanding virus replication, virus cell interactions, and the development of several virus-like particle (VLP) systems or rationally attenuated live-virus vaccine candidates (33–42). Live-attenuated RVFV vaccine candidates currently in trials were designed by deleting virulence factors (such as NSs and NSm) or by utilizing RVFV with naturally occurring deletions in virulence genes (43–46).

To this end, we sought in this study to generate recombinant SFTS phleboviruses that contain truncations in the NSs open reading frame (ORF) or have the NSs ORF replaced by a foreign gene of interest. Initial attempts made to recover recombinant SFTS phleboviruses lacking the NSs ORF failed to yield infectious virus despite repeated rescue experiments, including those where NSs was supplied in *trans* or where rescue experiments were performed in cells stably expressing SFTSV NSs. These data led us to believe that the SFTSV NSs protein or a sequence encoded within the NSs ORF was necessary for the recovery of infectious virus. Next, we generated a recombinant virus (rHB29NSsKO) in which the first two methionine residues were mutated to alanine and several stop codons were introduced in the coding sequence of NSs to abrogate protein expression. We demonstrate that in rHB29NSsKO-infected cells, NSs was not expressed and the virus could no longer form plaques in infected cell monolayers. In addition, rHB29NSsKO was unable to antagonize the production of IFN produced in response to infection and displayed faster replication kinetics in cells with a defective IFN response. Importantly, we demonstrate for the first time in the context of a virus infection that SFTSV NSs was responsible for the block in IFN production seen in SFTSV-infected cells. As it was evident that NSs protein was not essential for SFTSV replication but that some sequence within the NSs ORF was necessary for virus recovery, we mapped the 3′ ends of the N and NSs mRNAs produced in infected cells by 3′ rapid amplification of cDNA ends (RACE). We found that the N and NSs mRNAs overlapped, as previously noted for other phleboviruses, and that the 3′ end of the mRNAs extended past the IGR to terminate within the coding region of the gene encoded in the opposite orientation. We determined that the N mRNA terminates just downstream of a 5′-GCCAGCC-3′ nucleotide motif residing in the NSs ORF encoding amino acids (aa) 283 to 284 of the 292-amino-acid NSs protein. We establish that the presence and position of this motif are crucial for recombinant virus rescue, which can be presumably explained, as deletion of this sequence disrupts the correct termination of the N mRNA. With this knowledge, we generated viruses with internal deletions of NSs, viruses expressing only a 12-aa NSs peptide, and viruses that express enhanced green fluorescent protein (eGFP) or eGFP C-terminally fused to NSs from the NSs locus. The ability of the recombinant viruses to antagonize IFN production in response to infection was measured, and like rHB29NSsKO, viruses that did not express NSs could not block IFN production. We also confirmed that in all recombinant virus-infected cells, the 3′ end of the N mRNA terminated at the 5′-GCCAGCC-3′ motif, as shown for wild-type SFTSV. Finally, we demonstrated that the addition of the N mRNA termination motif to an S-segment-based minigenome/virus-like particle assay increased minigenome activity in the presence of the viral glycoproteins in donor cells and resulted in higher Renilla activity in recipient cells infected with virus-like particles.

The recombinant viruses generated and characterized in this study can now be assessed as potential vaccine candidates, while also providing new tools to study the biology of SFTS phlebovirus infection in mammals and arthropods both *in vitro* and *in vivo*.

## RESULTS

### Rescue of a recombinant SFTSV that does not express the nonstructural protein NSs but maintains S segment integrity.

**FIG 1:**
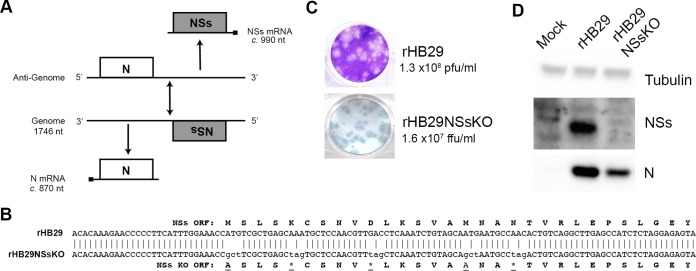
Recovery of a recombinant SFTSV that cannot express the virally encoded NSs protein. (A) Schematic depiction of the parental SFTSV S segment coding strategy, showing transcription of the NSs ORF encoding subgenomic mRNA from the antigenomic S RNA. (B) Alignment of the genomic S RNA coding sequences of rHB29 and rHB29NSsKO, showing the mutations made to abrogate the translation of the NSs protein from the NSs subgenomic mRNA (underlined). (C) Comparison of plaque or focus sizes of rHB29 and rHB29NSsKO on Vero E6 cells. Cell monolayers were fixed 5 days postinfection with 4% formaldehyde and stained with Giemsa solution or immunostained with anti-HB29 N antibody for a focus-forming assay; the average titers for 3 rescues are presented. (D) Vero E6 cells were infected at an MOI of 1 PFU or FFU/cell with recombinant viruses. At 24 hpi, lysates of infected cell monolayers were harvested and subjected to Western blotting. Blots were probed using anti-tubulin, anti-HB29 N, and anti-HB29 NSs antibodies.

Early reports showed that in SFTSV-infected cells, SFTSV NSs was found in virus-induced inclusion bodies (IBs), colocalized with the nucleocapsid protein, and was associated with viral genomic RNAs (25). Due to this observation, coupled with the failure of previous attempts to generate recombinant SFTSV that lacks expression of NSs (through the replacement of the NSs ORF with an ORF comprising a reporter gene), it was believed that NSs may play an essential role in virus replication (47). To address this issue and to take a different approach to deleting the whole NSs ORF from within the S RNA (depicted in Fig. 1A), we aimed to abrogate NSs protein expression by substituting the first two in-frame methionine residues with alanine residues (M1A and M16A) and introducing downstream stop codons at residues K5*, D10*, and N19* within the NSs ORF product (Fig. 1B). These mutations were inserted into the T7 transcription plasmid pTVT7-HB29S encoding the full-length cDNA of the SFTSV S segment, generating plasmid pTVT7-HB29SNSsKO. This plasmid, along with transcription plasmids pTVT7-HB29ppM and pTVT7-HB29ppL and expression plasmids pTM1-HB29N and pTM1-HB29ppL, was transfected into Huh7-Lunet T7 cells to generate rHB29NSsKO. The wild-type recombinant virus rHB29pp (herein known as rHB29) was rescued in parallel as a control. Titrations of rHB29NSsKO and rHB29 rescue supernatants were set up in duplicate; one set of titrated supernatants was processed for Giemsa staining and the other for immunostaining using an HB29 anti-N antibody. As previously documented, rHB29 readily formed easily distinguishable plaques visible by Giemsa staining (47). In contrast, rHB29NSsKO was detectable only in the immunofocus assay, producing foci of a size equivalent to that of the Giemsa-stained plaques of rHB29 (Fig. 1C). Three independent rescue experiments were performed, and the supernatants were used to infect Vero E6 cells to generate p1 stocks. Following passage, the mean titers of rescued rHB29 and rHB29NSsKO were 1.3 ×10^8^ PFU/ml and 1.6 ×10^7^ FFU/ml, respectively. To assess the synthesis of viral proteins in infected cells, Vero E6 cells were infected at a multiplicity of infection (MOI) of 1 and cell monolayers were harvested at 24 h postinfection (hpi) for Western blotting. Samples were probed with antibodies to detect SFTSV N and NSs proteins and alpha-tubulin as a loading control. N protein was readily detectable in both rHB29- and rHB29NSsKO-infected cells, whereas NSs was detected only in cells infected with rHB29, indicating that rHB29NSsKO does not express NSs during infection (Fig. 1D).

### Growth properties of recombinant SFTSV.

**FIG 2:**
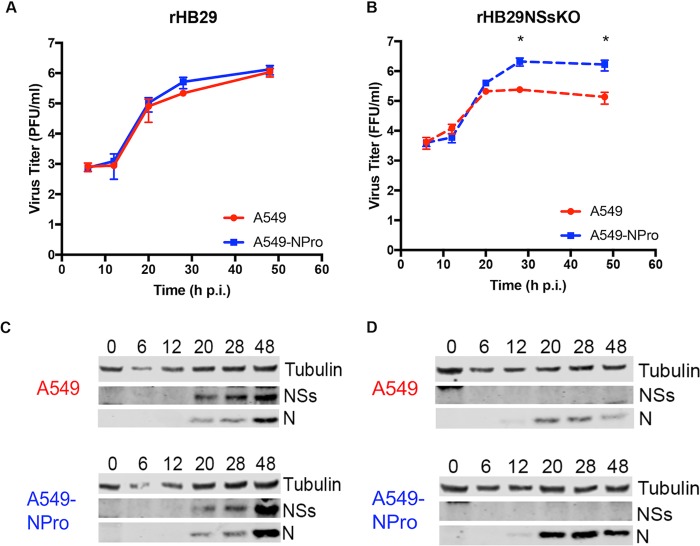
Growth properties of recombinant viruses. (A and B) Viral growth curves were determined for A549 or A549-NPro cells infected at a low MOI (0.1) with rHB29 (A) or rHB29NSsKO (B), and titers were measured by plaque assay or immunofocus assay as appropriate. Graphs show the average results of 3 experiments; all titrations were carried out at the same time (*, *P* < 0.05). (C and D) Western blot analysis of S-segment-encoded proteins from rHB29 (C)- or rHB29NSsKO (D)-infected cells. Cell extracts were prepared from the growth curve samples at the time points indicated, proteins were fractionated on 4-to-12% NuPage gels, and blots were probed with anti-HB29 N, anti-HB29 NSs, and anti-tubulin antibodies as indicated.

The growth properties of the recombinant viruses were assessed in human-derived interferon-competent (A549) and interferon-incompetent (A549-NPro) cells to evaluate the contribution of NSs to virus replication in the presence of the mammalian interferon response. A549 or A549-NPro cells were infected at an MOI of 1, and supernatants and cell monolayers were harvested at the time points indicated. An MOI of 1 was selected, since at lower MOIs, such as 0.1 focus-forming unit (FFU)/cell, rHB29NSsKO was unable to establish infection and replicate in A549 cells (data not shown). Viral supernatants were titrated on Vero E6 cells by plaque- and focus-forming assays. The parental virus rHB29 replicated efficiently, with similar growth kinetics in both A549 and A549-NPro cells, achieving peak titers of 1.1 ×10^6^ PFU/ml and 1.3 ×10^6^ PFU/ml, respectively (Fig. 2A). However, rHB29NSsKO replicated more efficiently in cells that had a defective interferon response (*P* > 0.05), reaching a peak titer 1 log higher in A549-NPro cells at 48 hpi (1.6 ×10^6^ FFU/ml in A549-NPro cells compared to 1.4 ×10^5^ FFU/ml in A549 cells) (Fig. 2B).

Western blotting of infected cell lysates collected throughout the time course revealed no substantial difference in the synthesis of the N or NSs proteins in either rHB29-infected A549 or A549-NPro cells. In both cases, accumulation of nucleocapsid protein was detected at 20 hpi and the protein continued to accumulate throughout the time course. NSs produced by rHB29 was also clearly detected from 20 hpi in both cell lines (Fig. 2C). In the case of the NSs knockout virus, N protein accumulated slowly in infected A549 cells and appeared to decrease at 48 hpi, reflecting the reduced replication kinetics observed in the growth curve (Fig. 2B). However, much greater accumulation of N protein was detected in the lysates from rHB29NSsKO-infected A549-NPro cells when the blots were exposed for the same length of time. Expression of NSs protein was not observed at any time point in either cell line infected with the NSs knockout virus (Fig. 2D). These data conclusively demonstrate that SFTSV NSs is not essential for virus replication in mammalian cells. The data also show that rHB29NSsKO replication is enhanced in cells lacking a functional IFN response compared to parental cells, where an IFN response is still intact.

### SFTSV NSs mediates IFN antagonism during infection.

**FIG 3:**
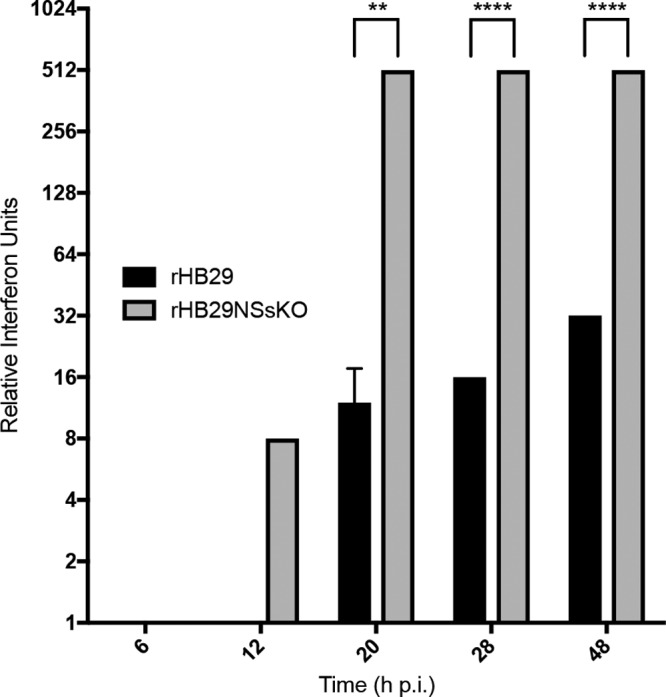
Interferon is induced to high levels in rHB29NSsKO-infected cells. Cell culture supernatants from viral growth curve samples were collected at the indicated time points. Samples from two experimental repeats were UV inactivated, and the numbers of relative IFN units were measured by a biological IFN assay. Error bars indicate the SD of the mean; ****, *P* ≤ 0.0001; **, *P* ≤ 0.01.

We next carried out a biological IFN assay on cell culture supernatants collected during the time course described in Fig. 2. Briefly, supernatants from recombinant virus-infected A549 cells were collected at the time points indicated (on Fig. 2A and B) and UV inactivated. Serial dilutions of the inactivated supernatants were then used to treat IFN-responsive A549-NPro cells for 24 h. Cells were then infected with IFN-sensitive encephalomyocarditis virus (EMCV) for 4 days to allow the cytopathic effect (CPE) to develop (48–51). Figure 3 shows that despite the fact that rHB29 encodes a potent IFN antagonist (22–27), rHB29 infection induced the production of interferon beginning at 20 hpi, reaching a maximum by 48 hpi. In comparison, rHB29NSsKO infection induced an IFN response early on (12 hpi), and the response increased rapidly for the remainder of the time course (*P* ≤ 0.0001). The data indicate that during parental virus infection, IFN is induced in response to infection and that this induction can be partially controlled by the expression of the viral NSs protein. These data also confirm that in the context of virus infection, SFTSV NSs acts as an IFN antagonist, in agreement with previous studies on bunyavirus NSs proteins (29, 31, 45, 52–56) and SFTSV NSs (22–27).

### Identification of the 3′ termini of SFTSV S-segment-derived mRNAs.

**FIG 4:**
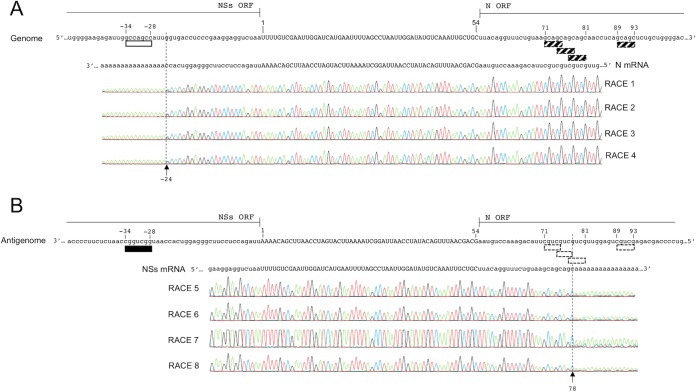
Mapping of the 3′ ends of HB29 N and NSs mRNA. Data are shown for four independent infections, RNA extractions, and 3′ RACE analyses of the N (A) and NSs (B) mRNAs of rHB29-infected cells collected 24 hpi. The sequences of the viral genomic or antigenomic S RNA and the N or NSs mRNAs are aligned. The intergenic region (uppercase) is shown from positions 1 to 54, and the N mRNA termination motifs 5′-GCCAGCC-3′ (white box) and 5′-GGCUGGC-3′ (black box) or NSs termination motifs 5′-GCUGC-3′ (white box, dashed outline) and 5′-GCAGC-3′ (hatched box) are shown. The N and NSs open reading frames are shown in lowercase. Arrows indicate the site of *in vitro* poly(A) addition.

The data described in the previous paragraphs demonstrate that NSs protein expression is not essential for SFTSV replication (Fig. 1). We next wanted to explore if there were any essential signals/sequences at the nucleotide level within the S RNA that might be preventing the successful recovery of a full NSs deletant virus through the deletion of the NSs ORF. To this end, we specifically wanted to identify the 3′ ends of the N and NSs mRNAs to establish the point at which the subgenomic mRNAs terminate within the viral RNA and pinpoint any motifs responsible for transcription termination. To facilitate this characterization, total cellular RNA was extracted from four independent replicates of rHB29-infected Vero E6 cells and 3′ RACE analysis was performed after *in vitro* polyadenylation with Escherichia coli poly(A) polymerase (E-PAP). Amplification of the 3′ end of the mRNA was achieved through reverse transcription-PCR (RT-PCR) with a primer specific for either the N or NSs mRNA and an oligo(dT) anchor primer. The PCR products were then sequenced (Fig. 4). In all four independent replicates, the 3′ end of the N mRNA was located at position −24 situated with respect to the start of the IGR, meaning that the N mRNA terminated within the antisense NSs ORF of the genomic RNA template (Fig. 4A). It was noted that this was upstream of a 7-nt sequence motif, 5′-GCCAGCC-3′, at positions −34 to −28, a motif similar to that first described for RVFV (12). The NSs mRNA terminated at position 78 within the antisense N ORF. Unlike that seen for the N mRNA, no 5′-GCCAGCC-3′ motif was observed upstream of the NSs mRNA termination site within the viral antigenomic RNA. However, there are several termination motifs identical to that seen with RVFV N and NSs mRNAs (5′-GCUGC-3′) located near the termination site at positions 71 to 75, 74 to 78, 77 to 81, and 89 to 93 (Fig. 4B) (11–13). As it was now apparent that a small portion of the 3′ end of the NSs coding sequence was utilized by the virus for N mRNA transcription termination, we next pinpointed the exact motif responsible for mRNA termination and examined whether this information would facilitate the successful recovery of viruses in which the coding sequence for NSs was replaced with another gene of interest (see below).

### Generation of recombinant SFTSVs with truncated or deleted NSs ORFs.

**FIG 5:**
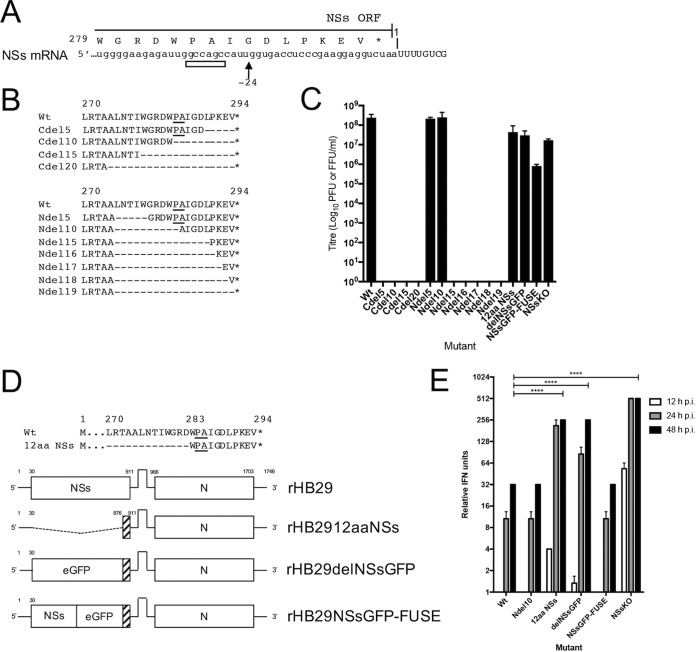
Generation of recombinant SFTS phleboviruses with truncated or deleted NSs ORFs. (A) Schematic depiction of putative N mRNA transcription termination signal and corresponding position in the NSs ORF. An arrow indicates the site of *in vitro* poly(A) addition. (B) NSs truncation mutants were made from either the C terminus of the NSs ORF product (upper panel) or N terminally from aa 274 to the stop codon in pTVT7-HB29S (lower panel). (C) The modified constructs illustrated in panels B and D were used in a reverse system to attempt to recover recombinant viruses. Briefly, rescue supernatants from transfected cells were collected at 5 days posttransfection and passaged onto Vero E6 cells. Monolayers were harvested at 7 days postinfection and titrated by plaque- or focus-forming assay on Vero E6 cells. Data are presented as an average result of three independent rescue experiments. (D) Modified pTVT7-HB29S cDNA constructs were generated expressing only a methionine and the last 11 aa of the NSs ORF product (a 96% deletion of the NSs coding sequence) or expressing a foreign gene of interest by fusing the last 11 aa of the NSs ORF product to the C terminus of the gene, as indicated in the diagram and also used in the reverse genetics system to recover recombinant viruses. (E) A549 cells were infected with recombinant viruses at an MOI of 1 PFU or FFU/cell. At the indicated time points, cell culture supernatants from infected cells were collected. Samples from three experimental repeats were UV inactivated, and the relative IFN units were measured by a biological IFN assay. Error bars indicate the SEM; ****, *P* ≤ 0.0001.

To assess the contribution of the putative N mRNA transcription termination motif (5′-GCCAGCC-3′) to the efficiency of recombinant virus rescue, a series of NSs truncation mutants was generated. First, the termination motif was aligned to determine where in the NSs amino acid sequence the motif resided (Fig. 5A). The termination motif was predicted to reside within the coding sequence for Pro-284 and Ala-285 of the NSs ORF. A series of NSs ORF C-terminal truncation mutations (Cdel5 to Cdel20) from the stop codon at aa position 294 or N-terminal truncation mutations (Ndel5 to Ndel19) from Ala-274 was generated by excision PCR of the cDNA plasmid pTVT7-HB29S. The cDNA constructs were used in our rescue system to recover recombinant viruses in three independent experiments and then passaged once in Vero E6 cells to generate working stocks of virus. Figure 5C shows that only recombinant viruses containing Ndel5 and Ndel10 were recovered at titers (2.2 and 2.5 ×10^8^ PFU/ml, respectively) similar to that of the parental rHB29 (2.4 ×10^8^ PFU/ml). Experiments to recover recombinant viruses containing NSs Ndel15 to Ndel19 deletion mutants did not yield any infectious virus in any of the independent experiments, indicating that the efficient/correct termination of the N mRNA was crucial for virus rescue. The recovery of the recombinant virus containing the Ndel10 mutant was surprising, as the Ndel10 mutation should have disrupted the 5′-GCCAGCC-3′ transcription termination motif. However, on examination of the nucleotide sequence of the truncation mutant at the truncation site, a nucleotide sequence of 5′-GCCGCC-3′ was generated in the same position as the authentic signal. Furthermore, 3′ RACE analysis confirmed that this motif is being used as an alternative transcription termination signal for the N mRNA, facilitating the recovery of the recombinant virus (Fig. S1). In contrast to the N-terminal truncations, none of the rescue experiments that include the C-terminal truncations yielded infectious virus. This was unexpected, as the Cdel5 mutant still contained the 5′-GCCAGCC-3′ motif.

Next, we wanted to confirm if the 5′-GCCAGCC-3′ motif was necessary for efficient virus rescue by truncating the NSs ORF within pTVT7-HB29S to encode a 12-aa sequence containing a methionine and the last 11 amino acid residues of the NSs ORF product (MWPAIGDLPKEV*; underlining indicates the amino acids in which the transcription termination resides) (rHB2912aaNSs). The sequence encoding this short NSs peptide contains the 5′-GCCAGCC-3′ nucleotide motif at the same number of nucleotides away from the N ORF stop codon (Fig. 5D). We once again used this plasmid in our reverse genetics system and passaged it once in Vero E6 cells, on all occasions recovering rHB2912aaNSs virus with an average titer of 4.4 ×10^7^ FFU/ml, almost 10-fold less than that of the wild-type virus (Fig. 5C). The successful rescue of rHB2912aaNSs indicated that the 36-nt sequence containing the 5′-GCCAGCC-3′ motif seemed to be necessary for virus rescue. Therefore, we generated a further set of recombinant S segment transcription plasmids in which the coding sequences of enhanced green fluorescent protein (eGFP) or NSseGFP (eGFP C-terminally fused to the HB29 NSs ORF) were cloned into the truncated NSs locus retaining the essential short peptide sequence described earlier (Fig. 5D). Recovery of the rHB29delNSsGFP and rHB29NSsGFP-FUSE recombinant viruses (with average stock titers of 3.0 ×10^7^ PFU/ml and 8.2 ×10^5^ PFU/ml, respectively) was achieved. Successful rescue of these viruses demonstrated conclusively that the NSs protein is not necessary for viral replication in infected cell monolayers and that the NSs ORF (apart from the sequence encoding aa 283 to 294) can be replaced with foreign genes of interest.

As NSs has been demonstrated to antagonize the mammalian IFN response, we wanted to see if our new recombinant viruses were able to prevent the production of IFN during infection. Briefly, A549 cells were infected with recombinant viruses at an MOI of 1 PFU/cell (rHB29, rHB29NSsNdel10, rHB2912aaNSs, rHB29delNSsGFP, rHB29NSsGFP-FUSE, or rHB29NSsKO), and infected cell supernatants were harvested at the time points indicated, UV inactivated, and subjected to a biological IFN assay as described in the previous paragraph. The data show that recombinant viruses containing intact or nearly complete NSs ORFs (rHB29NSsNdel10 or rHB29NSsGFP-FUSE) could antagonize IFN production as efficiently as the parental virus rHB29 at all time points tested. Conversely, recombinant viruses in which NSs expression was abrogated (rHB2912aaNSs, rHB29delNSsGFP, or rHB29NSsKO) induced large amounts of IFN from as early as 12 hpi, resulting in the production of significantly more IFN (*P* ≤ 0.0001) than that of the parental virus by 48 hpi (Fig. 5E).

**FIG 6:**
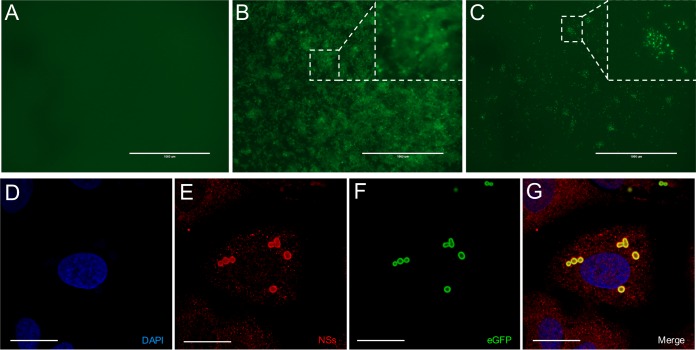
(A to C) Visualization of recombinant virus-infected cells. eGFP autofluorescence in Vero E6 cells mock infected (A) or infected with rHB29delNSsGFP (B) or rHB29NSsGFP-FUSE (C) imaged at 24 hpi. The boxes highlight differences in eGFP expression in infected cell monolayers. Bar, 1,000 μm. (D to G) To assess whether the eGFP signal detected in rHB29NSsGFP-FUSE-infected cells colocalized with NSs expression, Vero E6 cells were infected with rHB29NSsGFP-FUSE at an MOI of 1, and the cell monolayer was fixed in 4% formaldehyde at 24 hpi, followed by costaining with anti-HB29 NSs (red) or DAPI (blue) and examination for eGFP autofluorescence by confocal microscopy. Bar, 10 μm.

As we generated recombinant viruses that expressed eGFP or NSs tagged with eGFP that can form IBs, we wanted to examine the expression of the reporter genes in infected cells. Vero E6 cells were mock infected or infected with rHB29delNSsGFP or rHB29NSsGFP-FUSE at an MOI of 3 PFU or FFU/cell. At 24 hpi, cell monolayers were observed for eGFP autofluorescence on an EVOS FL microscope (Fig. 6A to C). As shown in Fig. 6, eGFP signal was seen diffusely spread through the monolayer of cells infected with rHB29delNSsGFP (Fig. 6B), compared to the mock-infected cells (Fig. 6A). The eGFP signal in rHB29NSsGFP-FUSE-infected cells showed the distinct punctate cytoplasmic staining associated with SFTSV NSs IBs described by many in infected cells (Fig. 6C) (22, 24, 47). The formation of the eGFP fusion inclusion bodies was further investigated by staining for NSs with anti-NSs serum and by visualizing 4′,6-diamidino-2-phenylindole (DAPI) (Fig. 6D), NSs (Fig. 6E), and eGFP (Fig. 6F) signal by confocal microscopy. As seen in Fig. 6G, the eGFP signal strongly colocalized with the NSs signal, indicating that the eGFP tag did not interfere with the formation of the NSs inclusion bodies present in infected cells.

### SFTSV N mRNA transcription termination occurs at the 5′-GCCAGCC-3′ transcription termination motif in S RNA of recombinant viruses.

**FIG 7:**
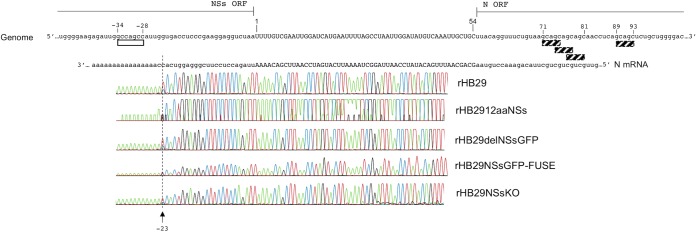
3′ RACE analysis confirms that the 3′ end of the N mRNA terminates at the same position in all recombinant viruses. Vero E6 cells were infected with recombinant virus rHB29, rHB1912aaNSs, rHB29delNSsGFP, rHB29NSsGFP-FUSE, or rHB29NSsKO at an MOI of 1 PFU or FFU/cell. At 24 hpi, cell monolayers were harvested; total cell RNA was extracted, and 3′ RACE analysis was performed. The sequences of the viral genomic S RNA and the N mRNA derived from recombinant virus-infected cells are aligned. The intergenic region (uppercase) is shown from positions 1 to 54, and the N mRNA termination motif 5′-GCCAGCC-3′ (white box) is shown. The N and NSs open reading frames are shown in lowercase. Arrows indicate the site of *in vitro* poly(A) addition.

3′ RACE analysis of infected cell RNA was used to confirm the utilization of the 5′-GCCAGCC-3′ motif as the signal for N mRNA termination within recombinant virus S RNA. Briefly, Vero E6 cells were infected with recombinant viruses at an MOI of 1 PFU or FFU/cell. Twenty-four hpi, total cellular RNA was extracted from infected cell monolayers, reverse transcribed using an oligo(dT) anchor primer, and subjected to 3′ RACE analysis as described above. Identical to the findings previously described in Fig. 4, the 3′ end of the rHB29 N mRNA was found to terminate upstream of the 7-nt 5′-GCCAGCC-3′ sequence motif at positions −34 to −28 (Fig. 7). Moreover, 3′ RACE data from RNA derived from cells infected with rHB2912aaNSs (expressing only the last 11 aa residues of the NSs ORF product from the NSs locus within the S RNA) showed that the 3′ end of the N mRNA was located at position −23 or −24, terminating in the same position as the parental virus rHB29. All other recombinant viruses containing the 36-nt sequence containing the 5′-GCCAGCC-3′ motif and encoding the terminal 11 aa of the NSs ORF product (rHB29delNSsGFP, rHB29NSsGFP-FUSE, or rHB29NSsKO) had N mRNA terminating at identical positions in the S RNA, upstream of the putative termination motif at positions −34 to −28. These data, especially the 3′ RACE data obtained for rHB2912aaNSs, strongly suggest that the 5′-GCCAGCC-3′ motif is the transcriptional termination signal for the N subgenomic mRNA (Fig. 7).

### S segment-based minigenome and VLP assays containing the N mRNA transcription termination motif show increased activity.

**FIG 8:**
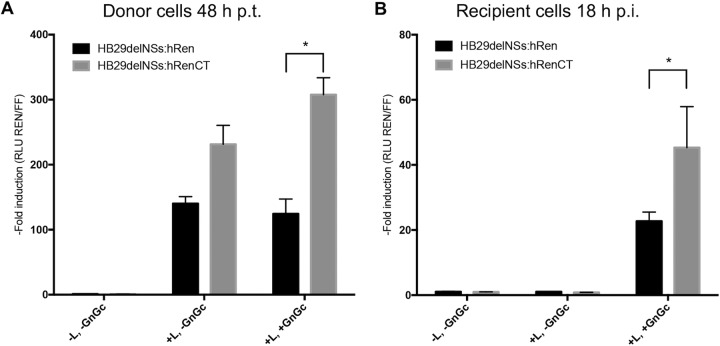
S-segment-based minigenome and VLP assays containing the N mRNA transcription termination motif show increased activity. (A) Effect of the addition of an 11-aa portion of the SFTSV NSs ORF product to the C-terminal end of the hRen gene on an S-segment-based minigenome with or without the inclusion of the viral glycoproteins in donor cells. Huh7-Lunet T7 cells were transfected with pTMFFluc (to measure firefly luciferase activity as an internal control of transfection efficiency and data normalization), pTVT7-HB29SdelNSs:hRen (T7-hRen), or pTVT7-HB29SdelNSs:hRenCT (T7-hRenCT) and pTM1-N, pTM1-L, or pTM1-GnGc where indicated. Empty pTM1 vector was used to ensure that the total amount of DNA used in each transfection was the same. Cell culture supernatants were harvested and nuclease treated, and firefly and Renilla luciferase activities were measured in transfected cell monolayers at 48 h posttransfection (h p.t.). (B) Luciferase activity in recipient cells. Nuclease-treated cell culture supernatants from the donor cells were adsorbed onto recipient Huh7-Lunet T7 cells for 1 h. Firefly luciferase and humanized Renilla luciferase were measured in VLP-infected recipient cells at 18 hpi.

Finally, we sought to decipher how the addition of the N mRNA termination signal affected the replication of viral ribonucleoproteins (RNPs) as well as the production of progeny virus particles, using an S-segment-based minigenome and virus-like particle assays. The minigenome system has been described previously (47). Briefly, Huh7-Lunet T7 cells which express T7 RNA polymerase were transfected with plasmids pTM1-N and pTM1-L and either pTVT7-HB29SdelNSs:hRen (T7-hRen) or pTVT7-HB29SdelNSs:hRenCT (T7-hRenCT; containing the 36-nt NSs coding sequence). To generate virus-like particles from transfected cells, plasmid pTM1-HB29GnGc expressing the SFTSV viral glycoproteins (Gn and Gc) was also included in the transfection mix where indicated on Fig. 8. At 48 h posttransfection, cell monolayers were harvested to measure firefly luciferase (FFLuc) and humanized Renilla luciferase (hRen) activity, and cell culture supernatants from donor cells (minigenomes with or without viral glycoproteins) were harvested and nuclease treated to eliminate any cDNA plasmid carryover (Fig. 8A). Both minigenomes showed increased activity over the negative control (−L, −GnGc) by 48 h posttransfection. However, inclusion of T7-hRenCT in the minigenome system led to a higher Renilla luciferase signal than when T7-hRen was used. The addition of the viral glycoproteins to the system (to generate VLPs) led to a statistically significant increase (*P* < 0.05) in minigenome activity in the T7-hRenCT-based minigenome compared to that of the minigenome containing just the hRen ORF. This difference is presumably mediated by the efficient production of virus particles in the T7-hRenCT system that can reinfect cells once produced, to boost Renilla luciferase signal. As this boost was not seen for T7-hRen-transfected cells, it is possible that although minigenome activity was detected, it was not enough to amplify the Renilla signal, a crucial step for the recovery of recombinant viruses in reverse genetic systems.

Renilla and firefly luciferase signals were measured in VLP-infected cells at 18 hpi. Only the supernatant of cells that had been cotransfected with pTVT7-HB29GnGc produced Renilla activity in recipient cells (Fig. 8B; +L, +GnGc), demonstrating that minigenomes were efficiently packaged into VLPs capable of infecting naive cells. The results also demonstrate that the nuclease digestion was sufficient to prevent plasmid transfer from donor to recipient cells (Fig. 8B; +L, −GnGc). Huh7-Lunet T7 cells infected with VLPs containing the T7-hRenCT minigenome had twice the activity of those containing T7-hRen (*P* < 0.05). These data indicate that the presence of the N mRNA transcription termination signal in T7-hRenCT leads to the efficient synthesis of N mRNA in T7-hRenCT-transfected cells, which results in increased minigenome activity in the donor cells. In the presence of the viral glycoproteins, this activity was amplified and could be efficiently transferred to recipient cells.

## DISCUSSION

The nonstructural (NSs) proteins of bunyaviruses are well documented as the main virulence determinant of infection and the primary virally encoded innate immune antagonist (27–32, 43–45, 50–52, 54, 55, 57–65). For this reason, research has been conducted to create and utilize NSs deletant viruses or viruses that contain natural truncations of the NSs proteins as potential live-attenuated vaccine candidates (34, 43, 44, 46, 55, 66, 67). In 2009, a novel tick-borne *Phlebovirus*, SFTSV, emerged in China. The virus caused severe disease in humans and had a high case fatality rate of 12 to 15% (15, 16). Upon the discovery of the virus, work began to investigate the contribution of SFTSV NSs to innate immune evasion and the pathogenesis of disease (22–27). The successful recovery of SFTSV entirely from cDNA clones was published in 2015 (47), with the aim of generating viruses lacking NSs proteins to facilitate pathogenesis studies and to develop novel vaccine candidates.

However, initial attempts to recover recombinant viruses in which the NSs ORF had been replaced with that of any foreign gene of interest before the publication of the reverse genetics system failed. Methodologies included direct rescues of NSs deletant viruses, trans-complementation of NSs in rescue experiments, and generation of NSs-expressing cell lines in which the rescue system could be used (data not shown). Work by Wu et al. (25) described a potential role for NSs in viral replication due to interactions of NSs with viral RNA and the nucleocapsid protein within the viroplasm-like structures present in infected cells. These data suggested that the recovery of recombinant viruses lacking NSs might not be possible due to an essential role of NSs in the viral replication cycle. However, data from minigenome experiments in which the NSs ORF had been replaced with the humanized Renilla luciferase gene indicated that RNP formation, replication of the RNPs, and subsequent Renilla luciferase expression was possible in the absence of NSs. In fact, the data showed that NSs inhibited minigenome activity when overexpressed in the system (47). With these data in mind, we hypothesized that the NSs protein itself may not be necessary for the formation of viral RNPs and hence virus replication. Here we show instead that the success of a given recombinant virus rescue could be determined by a signal or structure residing within the NSs nucleotide sequence.

We report the successful generation of recombinant NSs deletion mutant viruses through the mapping of the 3′ end of the N mRNA synthesized from the viral genomic RNA during infection. Surprisingly, even without synthesizing NSs (the well-documented interferon antagonist for SFTSV [22–27]), rHB29NSsKO could still replicate in A549 cells, albeit to lower levels (Fig. 2), perhaps suggesting that another virally encoded protein could have IFN antagonistic functions, an avenue of research worth exploring with future experimentation. To our knowledge, the data presented here are the first report that SFTSV NSs is responsible for biological IFN antagonism in the context of a virus infection (Fig. 3 and 5). This result highlights the importance of demonstrating the IFN antagonist activity of SFTSV NSs in the context of a virus infection. It was hypothesized that the reason for the enhanced pathogenicity of SFTSV was due to the antagonistic effect of NSs on the innate immune response. However, despite the fact that SFTSV encodes such a potent IFN antagonist, the synthesis of NSs in infected cells was not sufficient to efficiently block IFN production as a result of virus infection. Recent work has also shown that the severity of SFTS disease in SFTSV-infected patients correlates with high levels of IFN alpha (IFN-α), tumor necrosis factor alpha (TNF-α), granulocyte colony-stimulating factor (G-CSF), IFN-γ, macrophage inflammatory protein 1α (MIP-1α), interleukin 6 (IL-6), IL-10, interferon-inducible protein 10 (IP-10), and monocyte chemoattractant protein 1 (MCP-1), suggesting that in an *in vivo* setting, the ability of SFTSV NSs to antagonize the IFN response may be limited to type-I interferon induction and signaling (68).

**FIG 9:**
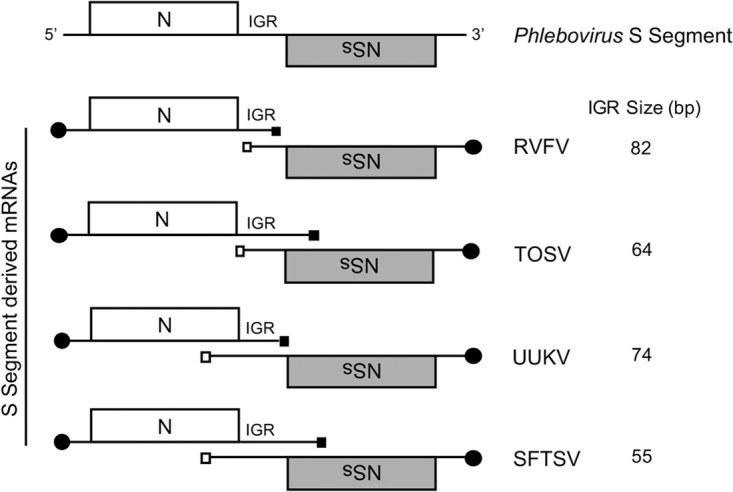
S-segment-derived mRNA termination of selected phleboviruses. A schematic representation is shown indicating where the 3′ ends of the N and NSs mRNAs of select phleboviruses map on the S RNA segment (not to scale). The size of each respective IGR is given. The 5′ cap structure on mRNA is depicted by a black circle. Black and white squares represent the N and NSs transcriptional termination signals, respectively. IGR, intergenic region; RVFV, Rift Valley fever phlebovirus; TOSV, Toscana virus; UUKV, Uukuniemi phlebovirus; SFTSV, SFTS phlebovirus.

From the data presented, it was clear that the NSs protein was not necessary for virus rescue or virus replication, even in cells with a functional IFN response. Our data also suggested that some part of the nucleotide sequence encoding NSs could be crucial for the recovery of recombinant viruses. Viruses within the Phlebovirus genus utilize an ambisense coding strategy to generate subgenomic mRNA transcripts expressing the N and NSs ORFs in opposite orientations, separated by an intergenic region (IGR) (3). Previous studies on several viruses, including RVFV (11–13), UUKV (6), and TOSV (14), have characterized and mapped the location of the 3′ end of the S-segment-derived mRNAs. For RVFV, both the N and NSs mRNAs were found to terminate within the IGR, downstream of a 5′-GCUGC-3′ motif (11–13). For UUKV, the N mRNA terminates in the IGR just before the end of the NSs ORF expressed in the opposite orientation, whereas the 3′ end of the NSs mRNA was mapped to a position within the N ORF expressed in the opposite orientation (6). Finally, the reverse situation was found to be true for TOSV, where the 3′ end of the NSs mRNA terminated just before the end of the N ORF and the N mRNA terminated at a position within the NSs ORF expressed in the opposite orientation, as summarized in Fig. 9. For SFTSV, we found that the 3′ end of both the N and NSs mRNAs mapped to positions within the coding sequence of the opposite gene (Fig. 4). Further mutational analysis determined that the termination of the N mRNA transcript was just prior to an upstream 5′-GCCAGCC-3′ motif, residing in the sequence encoding amino acids P-284 and A-285 of the NSs ORF product. We also confirmed that the nucleotide sequence 5′-GCCGCC-3′ in the same position as the authentic motif can be utilized as an alternative transcription termination signal in recombinant virus rHB29NSsNdel10.

For the C-terminal set of truncation mutants, no infectious virus was recovered in our reverse genetics system, even when amino acids P-284 and A-285 were present (Fig. 5B and D). However, in this case, the C-terminal truncation mutants resulted in the shortening of the distance between the N ORF stop codon and the transcription termination signal with each subsequent truncation. Work by Barr in 2004 on Bunyamwera orthobunyavirus (69) and by Lara et al. in 2011 on RVFV (13) proposed that there is a minimum distance between the N ORF stop codon and the transcription termination motif required for the RdRp to recognize the termination motif and detach, resulting in efficient N mRNA synthesis. Therefore, it is possible that the Cdel5 truncation moved the termination signal within this minimum distance, preventing the termination motif from being utilized by the transcribing RdRp, a hypothesis that will be explored in future studies.

Importantly, all recombinant viruses that encoded a full or partially truncated NSs (rHB29, rHB29NSsNdel10, and rHB29NSsGFP-FUSE) antagonize the production of IFN induced by virus infection as efficiently as the parental virus. Conversely, viruses that could not express NSs in infected cells (rHB2912aaNSs, rHB29delNSseGFP, and rHB29NSsKO) induced large amounts of IFN at late time points. rHB29NSsKO replication in A549 cells induced considerably more IFN production than the other NSs deletant viruses (Fig. 5E). This could be due to the production of an NSs mRNA that hybridizes with the S segment RNA to form double-stranded RNA (dsRNA), which could be sensed by melanoma differentiation-associated gene 5 (MDA-5) (70), an area to be elucidated further with time.

Finally, we wanted to assess how the presence of the 5′-GCCAGCC-3′ motif contributed to the ability to rescue recombinant NSs deletant viruses. This was assessed in the context of a virus-like particle formation assay to give a quantitative measure of RNP replication and packaging into virus particles. This process would be crucial for the efficient recovery of any attenuated or mutant recombinant viruses in a rescue system. In Fig. 8, we hypothesize that in both systems tested, the minigenome RNPs are formed and packaged into VLPs along with transiently expressed or encapsidated N protein from the donor cells, allowing for RNP replication and transcription and hence the detection of Renilla luciferase in the VLP-infected recipient cells. However, in T7-hRenCT-VLP-infected recipient cells, the minigenome RNPs would be able to efficiently synthesize additional N mRNA, which when translated into N protein will help increase the Renilla luciferase signal. This boost in signal, mediated by further N protein expression, translated from nascent N mRNA transcription in recipient cells, would be amplified over multiple rounds of replication and therefore be crucial in the ability to recover recombinant viruses in a rescue system.

In conclusion, we have demonstrated that SFTSV NSs protein is not essential for virus replication *in vitro*. Using an NSs knockout virus, we show that SFTSV NSs is responsible for antagonism of the innate immune response in the context of a virus infection. We have utilized 3′ RACE to map the 3′ end of the N mRNA, which allowed us to generate recombinant NSs deletant viruses that can be used for future studies on virus replication *in vitro* and *in vivo* and have the potential to be used as live-attenuated vaccine candidates to prevent spread of this important emerging pathogen.

## MATERIALS AND METHODS

### Cells and viruses.

A549 and Vero E6 cells (commonly used cell lines originally obtained from the European Collection of Authenticated Cell Cultures [ECACC] and previously described in references 48 and 71); Vero E6 from Institut Pasteur) were grown in Dulbecco's modified Eagle's medium (DMEM) supplemented with 10% fetal calf serum (FCS), and A549/BVDV-Npro (herein known as A549-NPro) cells (48) were grown in the same medium supplemented with 2 μg/ml puromycin (Melford Laboratories Ltd.). Huh7-Lunet T7 cells (72), which stably express T7 RNA polymerase, were obtained from R. Bartenschlager and were grown in DMEM supplemented with 2 mM l-glutamine, nonessential amino acids, and 10% FCS. All cell lines were grown at 37°C with 5% CO_2_ unless otherwise stated.

The SFTSV strain used in this study was a plaque-purified cell culture-adapted stock called Hubei 29pp (HB29pp) provided by Amy Lambert (CDC Arbovirus Diseases Branch, Division of Vector-Borne Infectious Diseases, Fort Collins, CO) (47) or a recombinant derivative of the virus generated by reverse genetics (rHB29). Working stocks of SFTSV were generated in Vero E6 cells by infecting the cells at a low multiplicity of infection (MOI) and harvesting the cell culture medium 7 days postinfection. Vero E6 cells were chosen to grow virus stocks due to the reported lack of a type-I IFN response. All experiments with SFTSV were conducted under containment level 3 (CL-3) conditions, approved by the UK Health and Safety Executive.

### Plasmids.

Plasmids for the recovery of SFTSV have been described previously (47). pTM1-HB29ppL and pTM1-HB29N contain the SFTSV HB29 L and N ORFs under the control of T7 promoter and encephalomyocarditis virus (EMCV) internal ribosome entry site sequence; pTVT7-HB29S, pTVT7-HB29M, and pTVT7-HB29ppL contain full-length cDNAs to the SFTSV strain HB29 antigenome segments flanked by T7 promoter and hepatitis delta ribozyme sequences.

Plasmid pTVT7-HB29SNSsKO (used to abrogate NSs protein expression while leaving the cDNA sequence intact) contains full-length cDNA to the HB29 S segment in which the cDNA sequence encoding the NSs ORF product has been modified to code for methionine-to-alanine substitutions at amino acid positions 1 and 16 and to insert stop codons at amino acid positions 5, 10, and 19. pTVT7-HB29S12aaNSs contains an internal deletion in the coding region of NSs (Δ2-282), leaving the methionine and last 11 aa of the NSs ORF product intact.

A series of individual S segment plasmids was generated based on pTVT7-HB29S in which the sequence encoding the NSs ORF product was C terminally truncated from the stop codon or N terminally truncated from amino acid 274. pTVT7-HB29SNSsCdel5, -Cdel10, -Cdel15, and -Cdel20 encode the HB29 NSs C-terminal deletions Δ289-293, Δ284-293, Δ279-293, and Δ274-293, respectively, while pTVT7-HB29SNSsNdel5, -Ndel10, -Ndel15, -Ndel16, -Ndel17, -Ndel18, and -Ndel19 encode the HB29 NSs N-terminal deletions from amino acid 274, namely, Δ275-279, Δ275-284, Δ275-289, Δ275-290, Δ275-291, Δ275-292, and Δ275-293, respectively.

pTVT7-HB29SNSseGFP-FUSE encodes an S segment that expresses NSs with eGFP fused at the C terminus. Transcription plasmids pTVT7-HB29SdelNSs:hRen, pTVT7-HB29SdelNSs:eGFP, and pTVT7-HB29SNSseGFP-FUSE were further modified by PCR-directed mutagenesis to add the last 11 amino acids of the HB29 NSs coding sequence (amino acids WPAIGDLPKEV) at the C terminus of the respective ORF product, immediately before the stop codon, resulting in the generation of plasmids pTVT7-HB29SdelNSs:hRenCT (T7-hRenCT), pTVT7-HB29SdelNSs:eGFPCT, and pTVT7-HB29SNSseGFP-FUSECT, respectively.

pTM1-HB29GnGc contains the HB29 M segment ORF under the control of the T7 promoter and encephalomyocarditis virus internal ribosome entry site sequence. All cDNA constructs were confirmed by Sanger sequencing. Oligonucleotides used to construct mutant segments can be found in Table S1 in the supplemental material.

### Virus titration by plaque- or focus-forming assays.

Virus titers were determined by focus-forming assays or by plaque assay in Vero E6 cells. Briefly, confluent monolayers of Vero E6 cells were infected with serial dilutions of virus made in phosphate-buffered saline (PBS) containing 2% FCS and incubated for 1 h at 37°C, followed by the addition of a Glasgow's MEM (GMEM) overlay supplemented with 2% FCS and 0.6% Avicel (FMC Biopolymer). The cells were incubated for 6 days before fixation and staining with crystal violet to visualize SFTSV plaques or use of focus-forming assays for recombinant viruses as described previously (47).

### Indirect immunofluorescence staining and live-cell imaging.

Vero E6 cells were grown to subconfluence on glass coverslips (13-mm diameter) and infected at a high MOI (3 FFU or PFU/ml) with recombinant viruses. At 24 hpi, the cells were fixed with 4% formaldehyde in PBS. Following permeabilization with 0.1% Triton X-100 and 50 mM glycine in PBS, proteins were detected using eGFP autofluorescence, rabbit anti-SFTSV NSs antibody (47), and secondary anti-rabbit Alexa Fluor 568 (Thermofisher). The coverslips were mounted on slides using Fluoromount-G with DAPI (eBioscience). Fluorescently labeled proteins were visualized using a Zeiss LSM-710 confocal microscope. Images of infected cell monolayers were also visualized for eGFP fluorescence using an EVOS FL cell imaging system (AMG; Invitrogen).

### Total cell RNA extraction and RT-PCR.

Vero E6 cells were infected at an MOI of 1, and total cellular RNA was extracted at 48 hpi using TRIzol reagent (Invitrogen). For reverse transcription-PCR (RT-PCR), 1 μg of total cellular RNA was mixed with a segment-specific oligonucleotide (Table S1), 0.5 mM 4× deoxynucleoside triphosphate (dNTP) mix (Promega), 40 U rRNasin (Promega), and 200 U Moloney murine leukemia virus (M-MLV) reverse transcriptase (Promega) and incubated at 42°C for 3 h. The resulting cDNA was used in PCRs with primers as described in Table S2, and the products were visualized by agarose gel electrophoresis. Products of the correct size were excised from the gel and purified using a Wizard SV gel and PCR clean-up system (Promega; A9282), followed by direct nucleotide sequencing of the PCR product.

### 3′ RACE.

3′ rapid amplification of cDNA ends (RACE) analysis was used to obtain both the 3′- and 5′-terminal sequences using strand-specific primers. Briefly, total cellular RNA was isolated from infected cells using TRIzol reagent (as described above), polyadenylated (Ambion; AM1350) for 1 h at 37°C, and then purified using an RNeasy minikit (Qiagen). Twelve microliters of polyadenylated RNA was then used in a reverse transcription reaction with M-MLV reverse transcriptase (Promega) and 100 μM oligo(dT) primer for 10 min at 65°C, followed by PCR using 0.3 μM 3′ RACE anchor primer and 0.3 μM segment-specific primer (Table A2) with KOD hot start DNA polymerase (Merck). Amplified products were purified on an agarose gel, and their nucleotide sequences were determined.

### Western blotting.

At different time points after infection, cell lysates were prepared by the addition of 300 μl lysis buffer (100 mM Tris-HCl [pH 6.8], 4% SDS, 20% glycerol, 200 mM dithiothreitol [DTT], 0.2% bromophenol blue, and 25 U/ml Benzonase [Novagen]), and proteins were separated on an SDS 4-to-12% gradient polyacrylamide gel (Invitrogen). Proteins were transferred to a Hybond-C Extra membrane (Amersham), and the membrane was blocked by incubation in saturation buffer (PBS containing 5% dry milk and 0.1% Tween 20) for 1 h. The membrane was reacted with anti-N or anti-NSs polyclonal rabbit antibodies (47) or an anti-tubulin monoclonal antibody (Sigma). This was followed by incubation with either horseradish peroxidase (HRP)-labeled anti-rabbit (Cell Signaling Technology) or anti-mouse (Sigma) antibodies. Visualization of detected proteins was achieved using Clarity ECL blotting substrate (Bio-Rad) and and a Bio-Rad ChemiDoc imager.

### Biological assay for interferon production.

A549 cells in 35-mm dishes were infected with 1 PFU or FFU/cell of the recombinant viruses and incubated at 37°C for the times indicated on Fig. 2A and B and 3. The medium was removed and treated with UV light to inactivate any virus (71), and 2-fold serial dilutions of the medium were applied to A549-NPro cells for 24 h. The cells were then infected with interferon-sensitive EMCV (0.05 PFU/cell), and the cells were incubated for 4 days at 37°C. The cells were then fixed with 4% formaldehyde and stained with Giemsa stain to monitor the development of CPE. The relative IFN units (RIU) are expressed as 2^*N*^, where *N* is the number of 2-fold dilutions that protect the reporter cells.

### SFTSV VLP assays.

The ability of S segment-based minigenomes to be packaged into virus-like particles (VLPs) was carried out using VLP assays, as previously described for several bunyaviruses (39, 73, 74). Briefly, subconfluent Huh7-Lunet T7 cells were transfected with 250 ng of the S segment-based minigenome-encoding plasmids (pTVT7-HB29SdelNSs:hRen or pTVT7-HB29SdelNSs:hRenCT), 125 ng pTM1-N, 25 ng pTM1-L, 250 ng pTM1-GnGc, and 25 ng pTM1-FFLuc as a transfection control. At 48 h posttransfection, the cell culture medium was clarified by centrifugation at 10,000 × *g* for 10 min and treated with 0.25 U/μl BaseMuncher (Expedeon) for 3 h at 37°C. Huh7-Lunet T7 cells were inoculated with the VLP-containing cell culture medium. At 18 hpi, the infected cell lysates were used to measure Renilla and firefly luciferase activities using the Dual-Luciferase reporter assay system (Promega).

### Generation of recombinant viruses from cDNA.

Recombinant SFTSVs were generated by transfecting 5 × 10^5^ Huh7-Lunet T7 cells with 0.1 μg pTM1-HB29ppL, 0.5 μg pTM1-HB29N, and 1 μg of each pTVT7-based plasmid expressing the wild-type or recombinant viral antigenomic segments, using 3 μl TransIT-LT1 (Mirus Bio LLC) per μg of DNA as the transfection reagent. After 5 days, the virus-containing supernatants were collected, clarified by low-speed centrifugation, and stored at −80°C. Stocks of recombinant viruses were grown in Vero E6 cells at 37°C by infecting them at an MOI of 0.01 and harvesting the culture medium at 7 days postinfection. The genome segments of the recovered viruses were amplified by RT-PCR, and their nucleotide sequences were determined to confirm that no mutations had occurred.

### Statistical analysis.

All data were analyzed using Prism 7 software (GraphPad) and are presented as the mean ± standard deviation (SD) or standard error of the mean (SEM). Statistical significance for the comparison of means between groups was determined by the Student *t* test. *P* values of ≤0.05 were considered significant (****, *P* ≤ 0.0001; ***, *P* ≤ 0.001; **, *P* ≤ 0.01; *, *P* ≤ 0.05).

## Supplementary Material

Supplemental material
