# Supplementary material for: Mapping of Transcription Termination within the S Segment of SFTS Phlebovirus Facilitated Generation of NSs Deletant Viruses
Source: J Virol. 2017 Jul 27;91(16):e00743-17. doi: 10.1128/JVI.00743-17 (PMC5533932; doi:10.1128/JVI.00743-17)
Supplement: Supplemental material [file supp_91_16_e00743-17__index.html]

Supplemental material 

# Mapping of Transcription Termination within the S Segment of SFTS Phlebovirus Facilitated Generation of NSs Deletant Viruses

## Supplemental material

- Supplemental file 1 -

  Fig. S1 (Mapping of the 3′ ends of rHB29NSsNdel10 N mRNA.)

  Table S1 (Oligonucleotides used for construction of plasmids used in this study.)

  Table S2 (Oligonucleotides used for 3′ RACE analysis.)

  PDF, 223K
