# Supplementary material for: Mapping of Transcription Termination within the S Segment of SFTS Phlebovirus Facilitated Generation of NSs Deletant Viruses
Source: J Virol. 2017 Jul 27;91(16):e00743-17. doi: 10.1128/JVI.00743-17 (PMC5533932; doi:10.1128/JVI.00743-17)
Supplement: Supplemental material [file JVI.00743-17_zjv999182825s1.pdf]

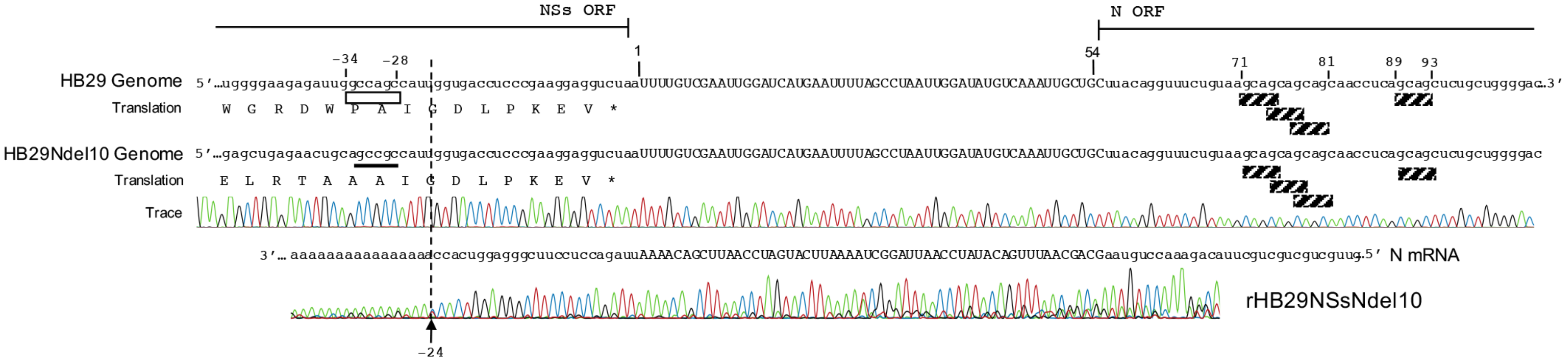

Fig S1. Mapping of the 3' ends of rHB29NSsNdel10 N mRNA. RNA extraction and 3' RACE analysis of the N mRNAs of rHB29NSsNdel10-infected cells collected 24 h p.i. The sequence of the viral genomic S RNA of rHB29 or rHB29NSsNdel10 and the N mRNAs are aligned. The intergenic region (uppercase) is shown from position 1 to 54 and the N mRNA termination motifs 5'-GCCAGCC-3' (white box) and the alternative transcription termination sequence 5'-GGCGGC-3' (underlined) are shown. The N and NSs open reading frames are shown in lowercase. Arrows indicate the site of in vitro poly(A) addition.

S Segment	Sequence 5'-3'	Purpose
HBNSsAUGKO+	cttcatttggaaacc**gct**tcgctgagc**tag**tgctccaacgttt**tag**ctcaaatctgtagca**gct**aatgcc**tag**actgtcag	Replace start codons with Alanine's and introduce stop codons in the NSs ORF within pTVT7-HB29S
HBNSsAUGKO-	gcctgacagt**cta**ggcatt**agc**tgctacagatttgag**cta**aacgttggagca**cta**gctcagcga**agc**ggtttccaaatga	
HB29NSsCtermdel+	taattttgtcgaattggatcatg	Serially truncate the NSs ORF within pTVT7-HB29S from the stop codon in to the NSs ORF. C del mutants
HB29NSsCtermdel5-	CAATTCGACAAAATTAgtcaccaatggctgg	
HB29NSsCtermdel10-	CAATTCGACAAAATTAccaatctcttccc	
HB29NSsCtermdel15-	CAATTCGACAAAATTAgatggtgttcagggc	
HB29NSsCtermdel20-	CAATTCGACAAAATTAtgcagttctcagctc	
HB29NSsdelNterm-	ggctgcagttctcagctcttcatcaaac	Serially truncate the NSs ORF within pTVT7-HB29S from position corresponding to aa 270 of the NSs ORF out towards the stop codon. N del mutants
HB29NSsdelNterm5+	GCTGAGAACTGCAGCCggaagagattggccagccattgg	
HB29NSsdelNterm10+	GCTGAGAACTGCAGCCgccattggtgacctcccgaag	
HB29NSsdelNterm15+	GCTGAGAACTGCAGCCccgaaggaggtctaattttgtcgaattg	
HB29NSsdelNterm16+	GCTGAGAACTGCAGCCaaggaggtctaattttgtcgaattg	
HB29NSsdelNterm17+	GCTGAGAACTGCAGCCgaggtctaattttgtcgaattggatcatg	
HB29NSsdelNterm18+	GCTGAGAACTGCAGCCgtctaattttgtcgaattggatcatg	
HB29NSsdelNterm19+	GCTGAGAACTGCAGCCtaattttgtcgaattggatcatg	
HB29GS12aaNSs+	ttagacctccttcgggaggtcaccaatggctggccaCATGGTTTCCAAATGAAGGGGGTTC	Truncate NSs in pTVT7-HB29S to encode only start codon and aa 283-294
HB29GS12aaNSs-	tggccagccattggtgacctcccgaaggaggtctaa	
TermSigAdd+	caaaaTTAGACCTCCTTCGGGAGGTCACCAATGGCTGGCTTGTACAGCTCGTCCAT	Add HB29 NSs aa 283-294 to pTVT7-HB29SdelNSs:eGFP or pTVT7-HB29SNSseGFP-FUSE
TermSigAdd-	CAAGCCAGCCATTGGTGACCTCCCGAAGGAGGTCTAAttttgtcgaattggatc	
TermSigAddhRen+	gtcaccaatggctggctgctcgttcttcagcacgcgctccac	Add HB29 NSs aa 283-294 to pTVT7-HB29SdelNSs:hRen
TermSigAddhRen-	ctcccgaaggaggtctaattttgtcgaattggatcatg	
eGFPforHNSs+	ggccagccattggtgacctcccgaaggaggtcATGGTGAGCAAGGGCGAGGAGC	To clone eGFP onto the c terminal end of

eGFPforHNSs-	ggctaaaattcatgatccaattcgacaaaaTTACTTGTACAGCT CGTCCATGCC	NSs in pTVT7-HB29S
HB29NSsLin+	ttttgtcgaattggatcatgaattttagcct	To linearise pTVT7-HB29S to allow for infusion cloning of eGFP
HB29NSsLin-	gacctccttcgggaggtcaccaatggctggcc	

Table S1. Oligonucleotides used for the construction of plasmids used in this study.

	Sequence 5’-3’	Genome Position (nt) (Genomic RNA)
S Segment		
N mRNA RACE	tatctgaagccacaaccaagaccctgatgg	1231-1202
N mRNA Seq	cagaagtttacaactccttcagggacc	1132-1106
NSs mRNA RACE	gagcctggagtccttcatggttacc	685-686
NSs mRNA Seq	tctattggactgttgggacttc	747-771
RACE-AP	gaccacgcgtatcgatgtcgac	N/A
Oligo-d(T)	Gaccacgcgtatcgatgtcgacttttttttttttttttv*	N/A

* v = A, C or G nucleotide

Table S2. Oligonucleotides used for 3’ RACE analysis.
